# COVID-19 advising application development for Apple devices (iOS)

**DOI:** 10.7717/peerj-cs.1274

**Published:** 2023-03-13

**Authors:** Saeed M. Alshahrani, Nayyar Ahmed Khan

**Affiliations:** Department of Computer Science, College of Computing and Information Technology, Shaqra University, Shaqra, Riyadh, Saudi Arabia

**Keywords:** Natural language processing, Advising, Healthcare, Electronic health records, iOS, Application

## Abstract

One of humanity’s most devastating health crises was COVID-19. Billions of people suffered during this pandemic. In comparison with previous global pandemics that have been faced by the world before, societies were more accurate with the technical support system during this natural disaster. The intersection of data from healthcare units and the analysis of this data into various sophisticated systems were critical factors. Different healthcare units have taken special consideration to advance technical inputs to fight against such situations. The field of natural language processing (NLP) has dramatically supported this. Despite the primitive methods for monitoring the bio-metric factors of a person, the use of cognitive science has emerged as one of the most critical features during this pandemic era. One of the essential features is the potential to understand the data based on various texts and user inputs. The deployment of various NLP systems is one of the most challenging factors in handling the bulk amount of data flowing from multiple sources. This study focused on developing a powerful application to advise patients suffering from ailments related to COVID-19. The use of NLP refers to facilitating a user to identify the present critical situation and make necessary decisions while getting infected. This article also summarises the challenges associated with NLP and its usage for future NLP-based applications focusing on healthcare units. There are a couple of applications that reside for android-based systems as well as web-based chat-bot systems. In terms of security and safety, application development for iOS is more advanced. This study also explains the block meant of an application for advising COVID-19 infection. A natural language processing powered application for an iOS operating system is indeed one of its kind, which will help people who need to advise proper guidance. The article also portrays NLP-based application development for healthcare problems associated with personal reporting systems.

## Introduction

The global pandemic outbreak emphasized the need for developing healthcare systems with the help of technology that can be helpful for humanity. The pressure on the public and healthcare system during the worldwide pandemic was very high (Legido-Quigley et al., 2020). Because of this, the healthcare units were about to collapse to provide better services to all the patients. Humanity suffered greatly due to the unavailability of medical resources and proper guidance during the global pandemic. The large scale of data remains to be available and analyzed during this period. Various innovative technologies exist. However, the use of technology was not in existence. The power of artificial intelligence (AI) came into the picture for the larger-scale data collected by the patients. The author expected the improvement of the public health system to occur with the help of these technologies driven by AI (DeSalvo et al., 2017). A subset of artificial intelligence technology is natural language processing. It resembles the analysis of large-scale datasets comprising texts collected from various channels. The electronic records from multiple hospitals, healthcare workers, small-scale dispensaries, medical literature, and social media sites. The information flowing from these locations is critical in discovering various factors related to COVID-19 patients (John, 2020). The global pandemic levers a lot of Information about various people’s health conditions (Venkatakrishnan et al., 2021).

**Figure 1:**
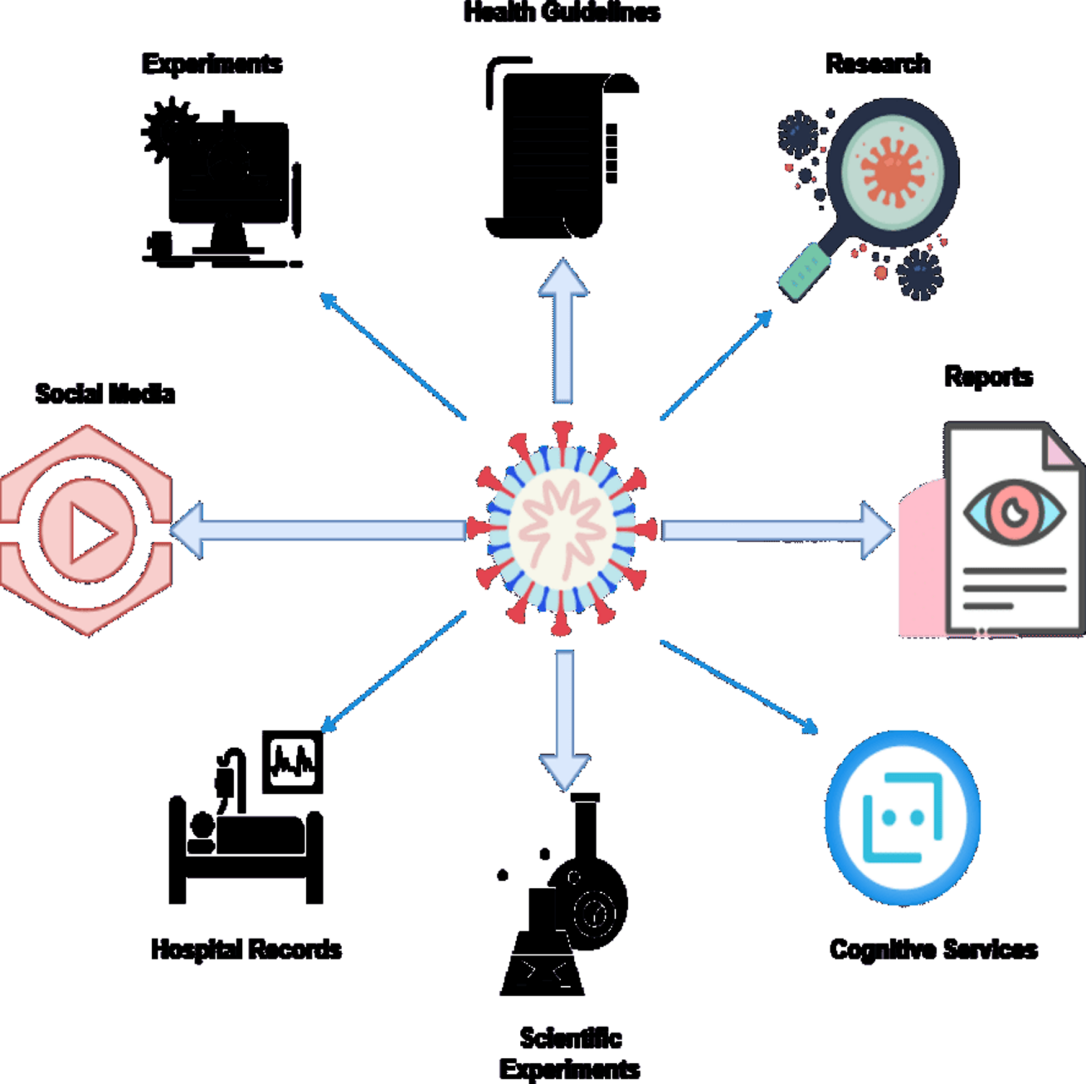
Data sources for text based analysis.

Analyzing these conditions is a must to handle the global vaccination process and develop proper drugs. One of the critical use cases of NLP is to identify a person’s symptoms and handle the situation accordingly. Khan & Albatein (2021) presented one of the fascinating models in which the authors constructed a WhatsApp advising bot. The application was able to take user responses with the help of NLP. Yet another compelling use case of NLP in healthcare applications relates to the sentiment analysis of the user. If a person faces health issues, his responses and replies relate to the psychological and sentimental state of mind. The analysis of these states is one of the influential factors that can lead to various suggested behaviors during the COVID-19 pandemic and such situations that might arrive soon. The application of NLP is a real-time analysis that the application can do to potential data flowing from various places. Once the data is connected, it can be analyzed to identify the associated problems and the probable solutions the author can create for this information (Yan et al., 2021). There are several data sources that the application can use for the identification of Information. Data flowed from these places across the pipelines and was analyzed with the power of natural language processing as shown in Fig. 1 below.

Many researchers and technocrats across the cross-domain of artificial intelligence and natural language processing addressed various topics. Some studies also present reviews related to using NLP (Guo et al., 2021; Chen & Liao, 2011; Hallak et al., 2020; Chatterjee et al., 2021). The AI-based methods empower various technical solutions for healthcare units.

**Figure 2:**
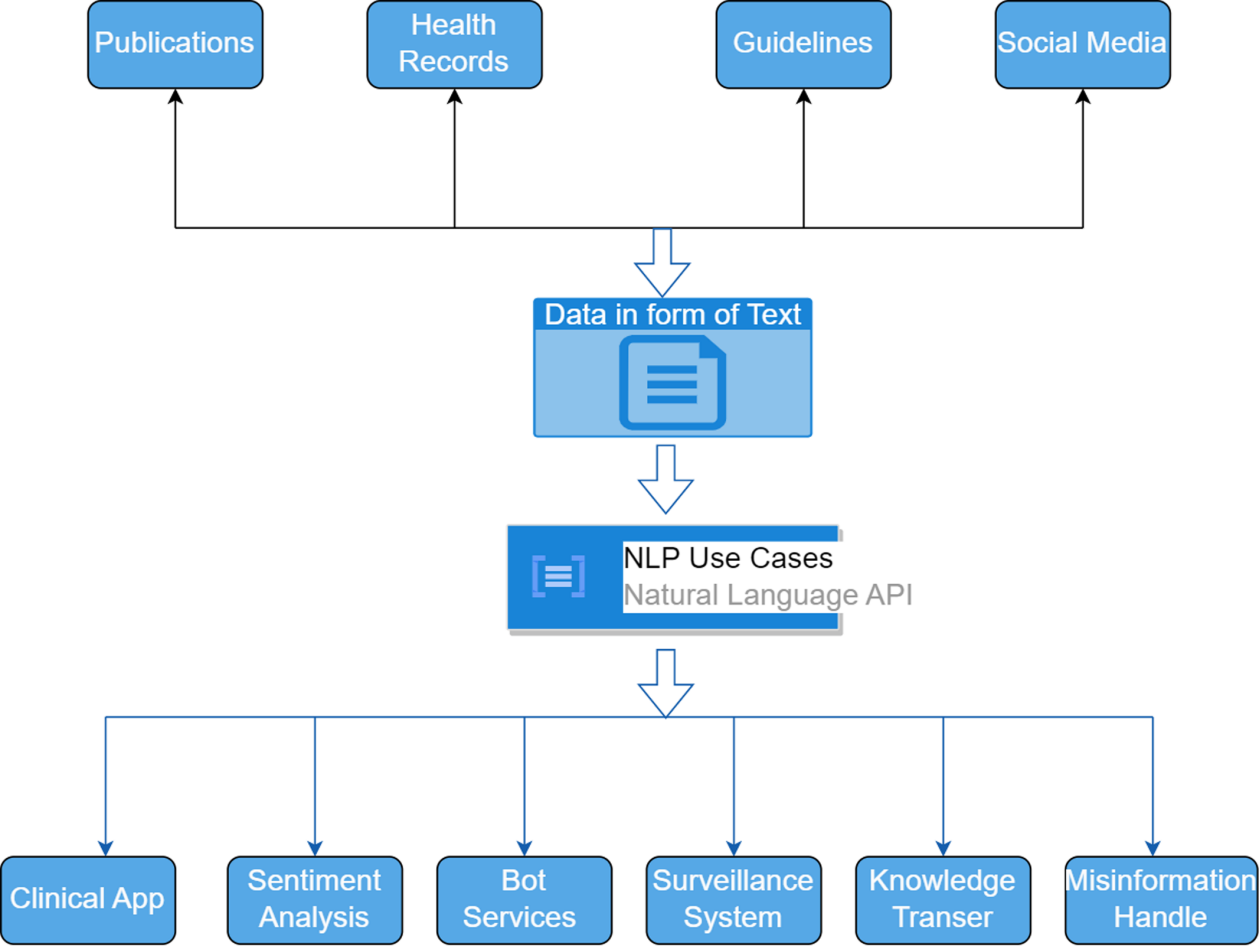
NLP use cases for healthcare data collected from various data sources.

The analysis of the data collected by the COVID-19 pandemic inputs from various sources, as shown in Fig. 1 above, is done with the help of NLP. Critical information from social media focusing on the sentiment analysis of human behavior reveals that an infected person needs an immediate response from healthcare facilities (Nham & Hong, 2022). Several deep learning applications were created and developed in the recent past (Shorten & Furht, 2021; Lalmuanawma & Lalrinfela, 2020; Islam et al., 2021; De Felice & Polimeni, 2020; Inés et al., 2021). The pre-trained NLP model discussed by various authors in several studies is of great importance. This study focused primarily on developing a COVID-19 advising application for iOS devices. Leswing (2020) reflects the fact that various applications have been rejected due to inconsistent behavior. Figure 2 above shows some of the use cases that NLP uses to collect data from various resources. The data for the healthcare units is usually collected from the health records of the hospitals. There are several Internet of Things (IoT) based devices also which stream the biometric data for the patients across various cognitive cloud services. The research based data from various nations are collected for medical situations and development of vaccines/medicines relative to an ailment *via* dashboards. It is also possible to collect data from various experimental research that is conducted by several medical and healthcare units.

The application is unique in the functionalities as specified in the abstract. Several applications are available across the App-store which focus on the COVID-19 support system. However there is a difference between the applications that are available and the proposed app. Several applications like Tawakkalna (Saudi Arabia) (https://ta.sdaia.gov.sa/en/index), WHO COVID-19 dashboard (https://covid19.who.int/), West Hills College district COVID-19 dashboard (https://westhillscollege.com/covid19/), etc. provides several features. But the unique feature proposed in this application are personal assistance for a patient. The app presented in the study uses ML and NLP to suggest users about their ailments. However this is found missing in all other similar applications hosted on the App-Store. However, the research focus in this article makes use of NLP to advise a user at a time when implicit help is required. Section 2 discussed various methods by which data from several healthcare units can be accessed. Section 3 describes the development of a model used by the machine learning packages offered by various agencies in the development. Section 4 describes the results and the use of NLP to handle and study the behavior of humans during ill conditions of health. The prediction systems consider various inputs, but the advising Application developed for iOS-based devices in this study focuses on the initial level of advising for a patient. This research focuses on the urgent situation in which a person suffering from an infection expects primary first aid and medicinal guidance.

1. Motivation: the mitigation for this research came from the fact that there are no applications for COVID-19 advice in the Apple Store. The authoring authorities have restricted several applications due to the occurrences of viruses or malicious codes in the application. The restriction is emphasized on the applications leading to the availability of real-time advisory/suggestive apps. The use of NLP in applications is increasing in all sectors. The development of applications empowered with artificial intelligence and NLP technology makes it possible for the healthcare unit to analyze a person’s health conditions. This study can use the collection of data in the form of electronic health records (EHR) and patient biometric information to treat health conditions (Topol, 2019). The main motivation is to empower the use of NLP and produce an application that can be used as an advisory companion for a person suffering from an infection.
2. Significance: the application supported in this research is accurate and of immense importance for the healthcare unit and the medical facilities across various countries. In the beginning stage of the application development, emphasis is given to developing the app for Saudi Arabia. However, the higher versions of the application are soon expected to be submitted over the global continents. The application eligibility to handle a patient’s health condition is one of the critical factors that lead to the significant achievement of the research (Locke et al., 2021).
3. Contribution: the key contributions that are expected to come out of this research are as below:
  • Designing an application for advising for COVID-19 infected patients.
  • Use of NLP to identify a patient’s condition and provide suggestive advice.
  • Collecting data sets from different persons related to their health conditions.
  • Analysis of the data collected from these patients to help the production of vaccination and other medical diagnostics.
  • Effectively use technology for handling critical conditions for patients undergoing an infection.

## NLP for Electronic Health Records

Scientists worldwide have found that to treat the diagnosis of a virus infection, and the prime requirement is the collection of Information related to the symptoms of various patients suffering from the infection. The adoption of health records produces a large volume of data for clinical research (Neuraz et al., 2020). A considerable amount of information is enough for analysis by the human investigator. However, the bulk amount of data from various healthcare and scientific research units is different from human intervention (Hall, Chang & Jayne, 2022). The role of NLP applications in such situations is very prompt and extensive. The collection of information from clinical data sources with the help of various sophisticated mechanisms is straightforward these days (Ohno-Machado, 2011). The medical literature and social media posts can be recognized and analyzed for further investigation across multiple pandemic states. Machine learning has also emerged as one of the most powerful technologies for handling data and analyzing Information for various healthcare units. The biometric factors of people suffering from an ailment can be collected in real-time and monitored very closely. Almalki et al. (2022) proposed a unique model that empowered edge computing in Association with blockchain networks to handle huge amounts of data. The cloud application provided the backbone of the company’s computational unit, and system passed on the Information to the decentralized peer to peer (P2P) network.

Using the clinical data collected with the help of NLP-based models can be done with various tools. There is an opportunity to develop such models during this global pandemic era (Ching et al., 2017). The main applicability of the data collected from multiple clinical sources relates to sentiment analysis and clinical diagnostics for any patient. For any disease domain, using NLP-based models can be of immense help. The diagnostics become fairly simple and effective after using cognitive services from various organizations. The expected knowledge from these ML models empowers the developers to create powerful applications to structure the learning. Self-reported Information from multiple patients and individuals in various formats such as audio, video, texts, tweets, blogs, *etc*. Barr, Ryan & Jacobson (2021) is critical to analyze the situation and probable symptoms of the disease. The phenotypes that are created after analysis of the Information submitted by the patient in the form of text can be used by various NLP models to handle the situation (Barr, Ryan & Jacobson, 2021). The collection of information is pretty simple with the help of technology. The analysis becomes eligible with the help of the NLP tools (Schöning et al., 2021). Various NLP models categorize the information into several levels of security Table 1. Below represents several NLP models that are used for handling the data from the records to predict the health status of a patient.

Depending upon the level of security as per the input given by the patient, the diagnostic can be done relatively simply. The NLP model is constructed for collecting data for COVID-19 symptoms, identifying the severity, and testing the diagnosis made by the healthcare professional (Wang et al., 2021). The data sets of these ML models can be further recycled to develop more sophisticated NLP systems. A combination of the two technologies empowers the prediction of the ailment a patient can be suffering from probably (Lybarger et al., 2021). The summary of the information available from various healthcare facilities related to the entire diagnosis of a patient ailment can also be treated as one of the most important inputs towards any NLP model (Prada Muñoz & Sutta Serrano, 2021). A further improvement in the diagnostic procedure for such ailments with similar symptoms can be repeated depending upon the medical practitioner’s guidance. Identifying any infection can have further clinical trials or expensive laboratory-related surveillance methods.

It can bypass directly providing proper treatment and starting the healing procedure without delays. With the various improvement in the technology and NLP techniques, the real-time self-reporting fall various symptoms of COVID-19, along with the extraction of accurate information results, can be concluded (Chapman et al., 2020). The prediction power of the application can be improved and modified related to the inputs collected from various individual users or healthcare facilities (Chapman et al., 2020). The information collected from these facilities can be further investigated and analyzed at various research labs to manufacturing proper vaccination or drugs that can reduce the disease severity. Several issues are also considered in the information received from various health sources, including mental health hazards (Fernandes et al., 2021). The privacy concern for individual information is of the utmost priority. However, Alangari et al. (2022) suggested a secure method for maintaining privacy with the help of blockchain networks. The data can be submitted to more secure channels to avoid any privacy hindrance to an individual patient. Several other models to maintain a patient’s privacy and handle the notes related to the diagnosis by a medical practitioner make use of NLP technology as well.

**Table 1:** Table models used to handle data from electronic records.

**Employed model**	**Data source**
Li et al. (2021)—random forest	32,000 reports from MRI and CT scans
Schöning et al. (2021)—NLP Rule-based pipeline	6,200 patient data analysis
Lybarger et al. (2021)—BERT with attention	1,450 diagnosis notes for COVID-19 tests.
Wang et al. (2021)—NLP rule based pipeline	Data-sets with 50+ posts on social networks
Cury et al. (2021)—Keyword extraction NLP (unsupervised)	450,000 patients report analysis (unsupervised learning)
Obeid et al. (2020)—Word2Vector translation	6,800 patients data for infection
Barr, Ryan & Jacobson (2021)—NLP entity recognition	Audio/video recordings analysis
Fernandes et al. (2021)—Multi class logistic regression model	1,737 adult discharged patients analysis
Chapman et al. (2020)—NLP Rule-based	Clinical data from the VA corporate data warehouse

## Development of the NLP Model for the iOS Application

**Figure 3:**
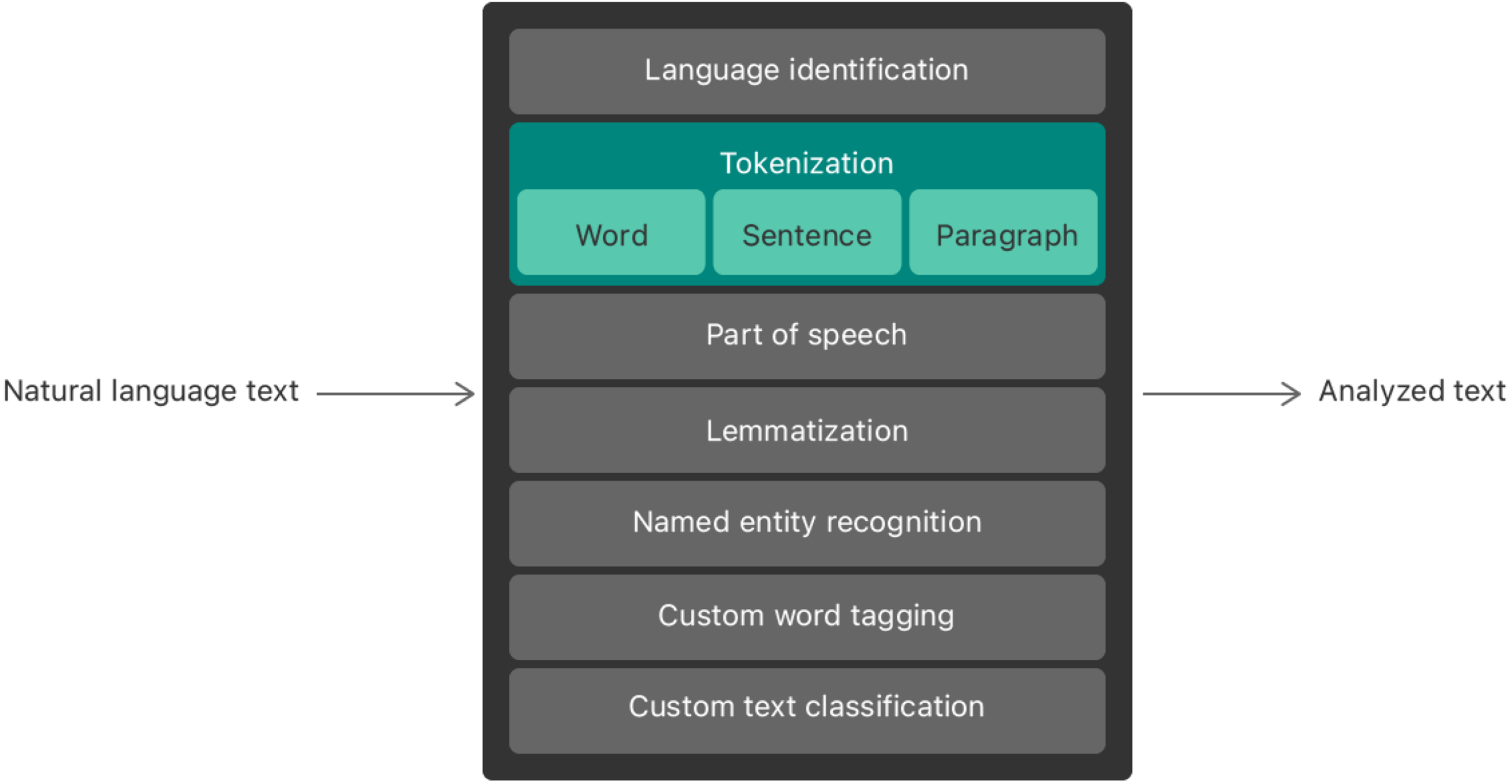
NLP components—apple natural language processing framework.

The application development suggested in this research focuses entirely on the use of technologies and frameworks suggested by iOS development. The development has been completely done with the help of NLP techniques originating from the natural language Toolkit of Apple. In this research, the Application is created with the help of the Core ML 3 framework. Several exciting techniques for language processing can be done with the help of the framework. Furthermore, various predefined ML models are supported by this framework to make the development procedure very easy and effective as shown in Fig. 3.

### Requirements for the development

1. XCode: the development environment for designing IOS applications is Xcode. It is not an integrated development environment in which predefined libraries for development are available. For developing the application in this study, Xcode version 14.2 is used.
2. Tokenization: the application works in two stages in which the user input is taken, and they are tokenized with the help of the machine learning (ML) libraries for NLP. The tokenization procedure breaks the peace of text into meaningful information or raw units. These units are broken depending on the sentence length submitted by the user. Then, the tokenized import is split into various fragments and stored as an array.
3. NLTokenizer: the natural language library provided by the Core ML3 package for identifying the Information submitted by the user in the form of text language is called NLTokenizer. The raw sentences are passed to this class, where it is further processed and split into various tokens. Then, these tokens are analyzed for the user answers and based on the inputs, and the Information is segregated.
4. Lemmatization: it is a procedure in which the raw input given by the user is trained and passed through various input parameters and processed for normalization. The normalization takes place to make meaningful words out of the different formats given as input by the user. The user may probably make smaller smelling mistakes or tense problems that the system can resolve in this stage. In developing the application for this research, we use the NLTagger class to identify the English word’s lemma. All the named entities are recognized, and the speech is tagged for further analysis by the application.
5. Localization: the language localization for the application is also possible with the help of the NLP package available in the core ML3 libraries. But at this point, we focus mainly on the English version of the application irrespective of the geographical localization. The application is expected to replicate various demography depending upon the popularity and usage across the countries. The use of NLLanguageRecognizer from the language Toolkit will be motivated for that cause.

**Figure 4:**
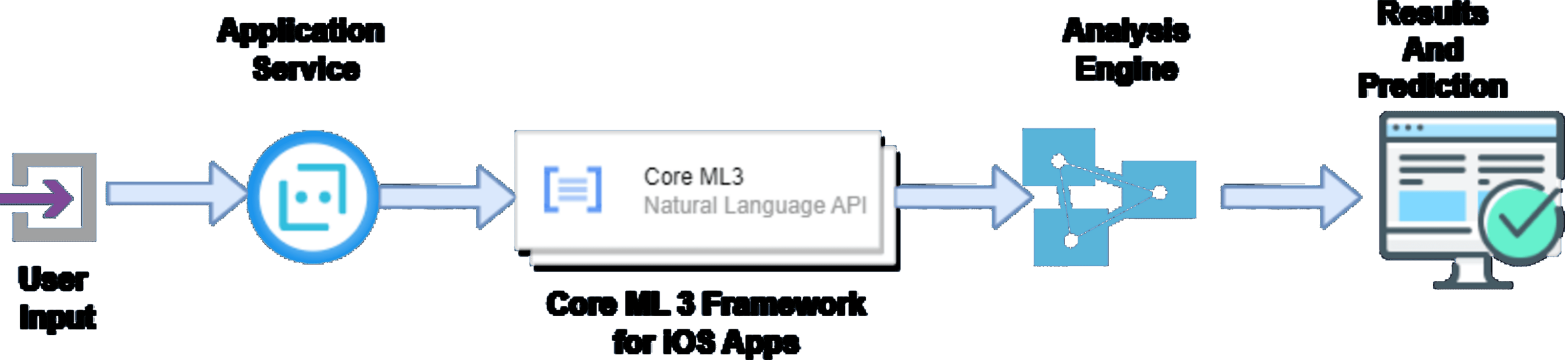
Working model for the proposed application.

The suggested model in this study works in several stages, as shown in the Fig. 4. First, the user gives the initial inputs. These inputs are based on various questions the application will ask the user. These questions will have a workflow dependent upon the ML model used in this application development. Once the application service takes all the inputs from the user, the user will send them to the ML language analysis framework. This framework is available along with the Core ML3 framework given by the eye development environment. The libraries and functions used for this development comprised two major stages. Tokenization and lemmatization take place at this level, and the final information, broken up into different fragments, is sent to the analysis engine. Depending upon the answers given by the user, the analysis engine decides the similarity of the disease a patient is suffering from. In the case of this application, the questions asked at the initial user input level of the application tend to identify the condition of a user while he is undergoing an infection. The use of Apple iOS ensures the security of the application from any unauthorized access or bugs. The design of the iOS is also effective to handle the code and avoid operating system issues.

The fragmented questions in this study undergo a flow analysis based on the answers suggested by the user. Once the analysis is completed, the prediction for the possible problem that the patient is facing is given. The application assures that the inputs given by the user are natural language. They are processed and based on his answers, and the prediction is made. The analysis engine is programmed in a way where the user answers are considered. These results are analyzed and finally reported back to the user. Based on the proper definitions and symptoms published by the World Health Organisation, the analysis engine protects the nature of similarity and its possible remedy.

The analysis engine is the core of the application and its acceptability which is capable of identifying the user results and categorizing the severity of the infection. Depending upon the user inputs, the entities are recognized by the analysis engine as per the answers. These entities decide the severity of the infection. Yet another important feature that the Application produces is a patient’s sentiment analysis. The prediction of positive, negative, or neutral sentiments is made with the help of the same natural language processing.

(1) \begin{eqnarray*}\text{Sentiment Score}= \left\{ \begin{array}{@{}ll@{}} \displaystyle Positive&\displaystyle x\gt 0\\ \displaystyle Negative&\displaystyle x\lt 0\\ \displaystyle Neutral&\displaystyle x=0 \end{array} \right. \end{eqnarray*}

The above equation supports the understanding of the Sentiment Analysis Score based on which a user sentiment can be studied for the prediction of the critical nature of the problem he is facing. The critical situation will have more value of the negative sentiment score and the non critical nature will be represented by a more positive value of the score. However, the neutral value of the score will result in a midway situation for the prediction.

**Figure 5:**
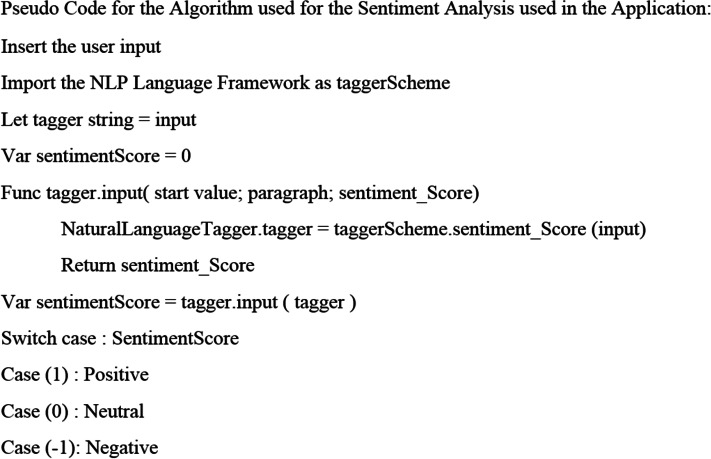
Pseudo code for the proposed algorithm.

The algorithm depicted in Fig. 5 performs the sentiment analysis for the user input given at any stage in the Application. A positive value of the sentiment score states that the user is hopeful about his condition and requires minor attention towards his medical treatment. In this case, the probability of getting recovered and rescued will be the highest. A neutral value for the sentiment analysis results in the user’s inability to express the complete symptoms and requires quick attention from the medical healthcare units. Further, a negative value of the sentiment analysis score assures that the user is not in a proper situation and urgent medical attention is required. The answers given by the user are predicted for his current situation related to this infection. The value of the sentiment score is significant to protect the nature of the issue that the person is facing. However, the NLP analysis, with the help of the ML model, makes it possible for medical healthcare professionals to manage the inputs the user gives for his diagnosis. The symptoms and the Information shared by the user will be classified with the help of the ML model, making use of the core ML3 framework used for iOS development. This Information is helpful for further analysis that the application can do at various diagnostic laboratories and research centers.

### Screenshots for the application

**Figure 6:**
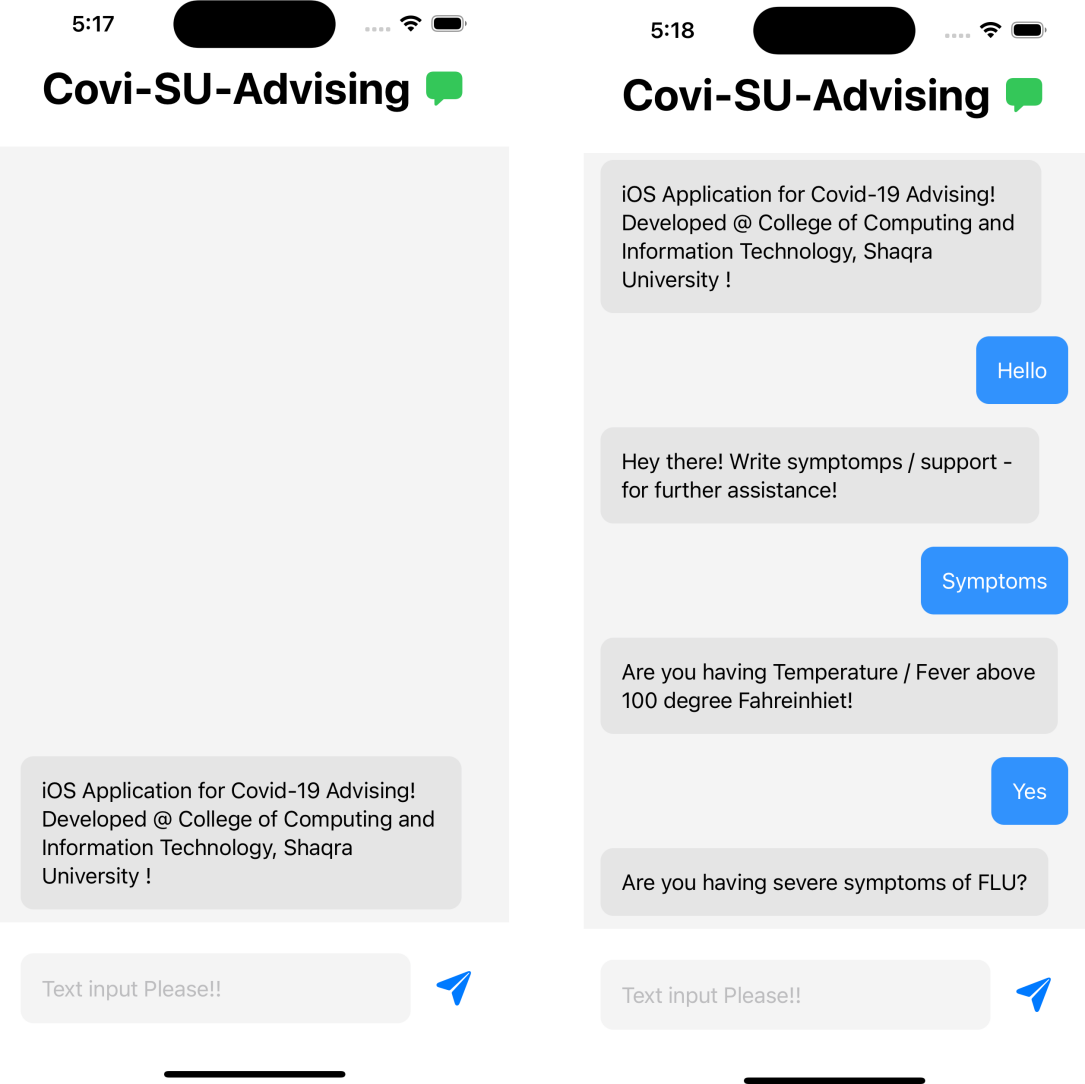
Working screen for the COVID-19 advising application.

**Figure 7:**
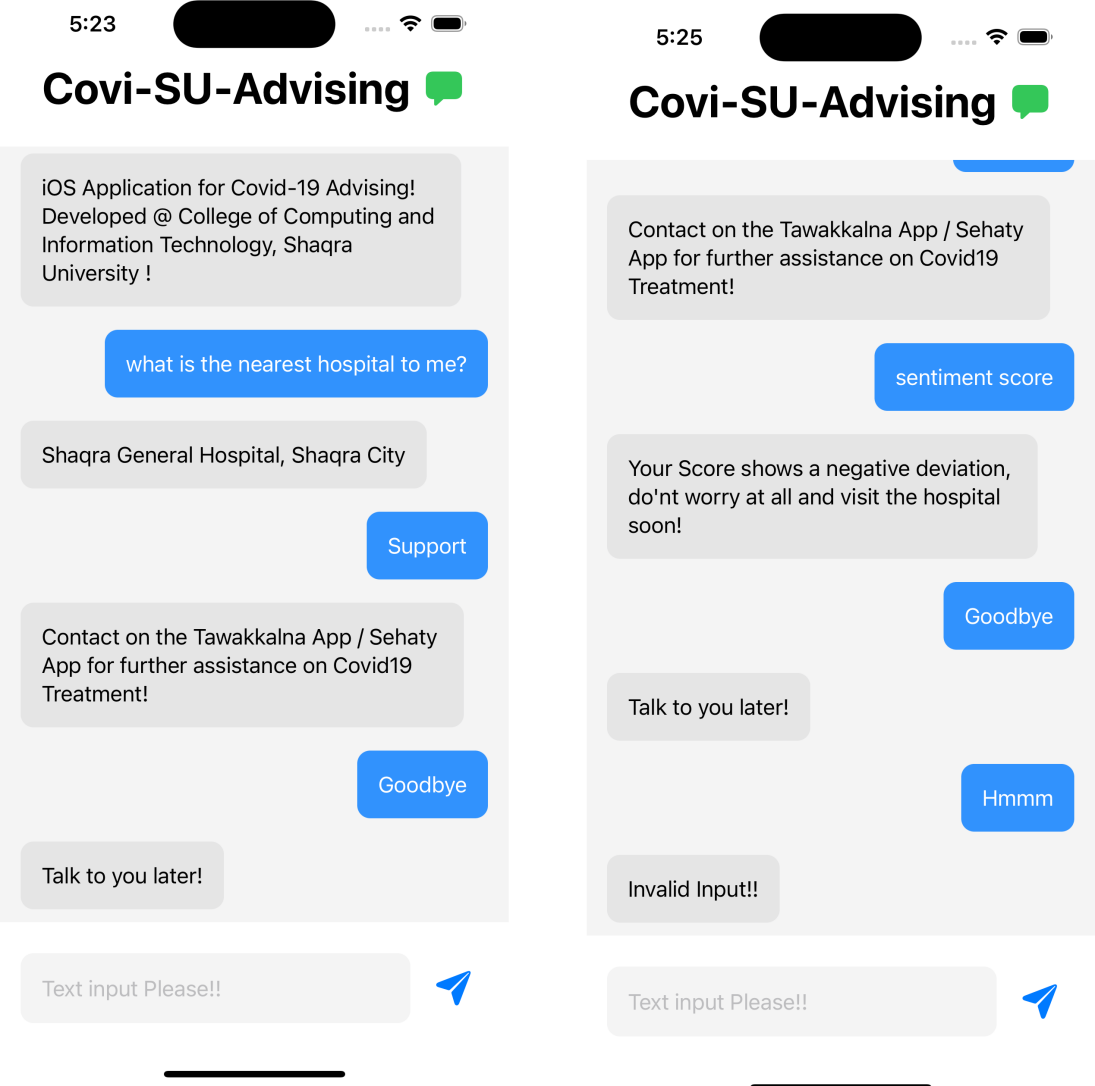
Sentiment analysis screen for the application.

Figures 6 and 7 represents the basic screens of the application developed as a part of this project. The application runs on any iOS devices running with the latest operating system. The application is developed with Swift programming language. The natural language processing framework by Apple Development is used in the application development stages.

## Discussion

During the global COVID-19 pandemic, governments worldwide led various impositions to control the spread of the infection. Problems associated with personal freedom, movement restrictions, the spread of fake information, and various other uncertainties and irregularities were observed during this time. The information was restricted to a demonstrator group of people only. It has been reported by Pfefferbaum & North (2020) that many people suffer from mental and psychological problems. The distress faced by a large population became the primary basis of public health concerns. The application can do the analysis of the sentiments as well as the emotions very well with the help of NLP-based applications. The power of NLP can handle information from various platforms to recognize the real-time situation of human beings. The application suggested in this study focus on the identification of the basic ailments of a patient.

Several NLP models comprise massive datasets that can analyze up to 200 emotions in English-language (Li et al., 2020). Real-time social media posts and Information from blogs can be constructive in finding the correlation between mental health and the present situation of the population. A large group of people’s trauma can be managed and identified with the underlying power of NLP. Real-time monitoring of such problems can be very helpful in providing a vital solution and reducing the negative impact on the remaining society. The application can do medical health surveillance with the help of such applications that focus on the identification of the sentimental value of a human.

The second important feature that the above application integrates is understanding the behavior of a patient’s health. To avoid the spread of infection, the model can identify the behavior of a human being based on his inputs and location. The application renders the location to prohibit the further spread of infection from one place to another. The responses given to the user in the application are forwarded to the machine learning algorithm to predict the nature of the illness and calculate the sentiment score. The value of the sentiment score decides the prediction capability and priority of medical attention. A negative value of the sentiment score provides a strong reason to monitor the patient. The real-time answers from a patient provide quick, actionable insight. The availability of open-access publications and scientific findings makes it possible to derive a conclusion with the help of the analysis engine.

## Conclusion

The use of natural language processing is increasing in various sectors of the computational systems. The language understanding is done with the help of inputs from various data sources. For the patients who are having health issues (related to COVID-19) this article produces a complete description of developing an application. The application runs in two phases in which the first phase takes input from the patient who is using the application. The patient can interact with the application in real world language and the replies for the patient queries are given. The application uses the power of natural language processing to respond back to the patient. The second phase of the application checks the patient sentiment score to find out his mental status during the use of the application. The results of the sentiment score are used to predict the emergency circumstances of the patient health. Based on the above observations the application advises the user to take necessary steps for the better health. The application designed for this study ensure the use of the Core ML3 libraries by Apple resources which empowers the application for the real time language processing. The application is developed to ensure that an advising application is available to help the iOS users for the advising during health conditions, particularly COVID-19. As for the future work, the application can be generalized to various ailments existing across the medicinal healthcare facilities.

## Supplemental Information

10.7717/peerj-cs.1274/supp-1 Supplemental Information 1 Raw data code in Swift programming language for the application The code requires XCode version 14.2 to run the simulation on a Mac device with at least an M1 chip integrated for processing.

10.7717/peerj-cs.1274/supp-2 Supplemental Information 2 Testing code with test cases The application was created for advising COVID-19 patients and was tested in the Apple XCode Development Environment.

10.7717/peerj-cs.1274/supp-3 Supplemental Information 3 Raw Data table of Comparison The Table contains the RAW DATA while comparing the application with the other competitor applications.
The application developed in this study is one of the unique applications with a new and noble idea. We have developed the application for iOS Platform and all the competitive applications are checked as per their popularity with the exisiting application that we developed.
The application is have a comparison part explained in one more file attached with the supplementary files (Comparison Table.pdf).
A Comparison is made with the features proposed by the application under this study to the existing applications in the market. The use of Natural Language Processing and Artificial Intelligence is a key feature that the proposed application adapts. This empowers the application to provide the users more efficient mechanism to help the patients who might have a health issue with the Covid19 infection. The application also suggests various features like the nearest hospital address. Table1. Shows the comparison of the Application with existing competing applications. The features are listed in the first column and 5 applications are benchmarked with the current proposed application.
It is worth mentioning that the application proposed in this research is the first of its kind application for the Covid19 patient advising. The application is very useful for those who are having a minor infection or extreme health conditions. The NLP based system powered by artificial intelligence helps the user to decide the prioritized action to be taken while he is suffering from any infection.
The application can be generalized for various other types of diseases in the near future.
1. Coronavirus (COVID-19), West Hills College District, https://www.whccd.edu/covid19/, https://westhillscollege.com/covid19/
2. 19 Advisor, App Store, https://apps.apple.com/us/app/covid-19-advisor/id1503546660
3. iOS 13.7: Apple’s New COVID-19 Contact Tracing System Explained.
https://www.forbes.com/sites/kateoflahertyuk/2020/09/02/ios-137-apples-new-covid-19-contacttracing-system-explained/?sh=3645bf1e6d2e
4. Tawakkalna, Covid Apps on Google Play, https://play.google.com/store/apps/details?id=sa.gov.nic.tawakkalna&hl=en_US&gl=US
5. WHO launches COVID 9to5Google, https://9to5google.com/2020/04/11/world-health-organization-who-covid-19-app/

## Additional Information and Declarations

### Data Availability

The following information was supplied regarding data availability: The code for the development of the application is available in the Supplementary Files.

## References

[ref-1] Alangari S, Alshahrani SM, Khan NA, Alghamdi AA, Almalki J, Al Shehri W (2022). Developing a blockchain-based digitally secured model for the educational sector in Saudi Arabia toward digital transformation. PeerJ Computer Science.

[ref-2] Almalki J, Al Shehri W, Mehmood R, Alsaif K, Alshahrani SM, Jannah N, Khan NA (2022). Enabling blockchain with IoMT devices for healthcare. Information.

[ref-3] Barr PJ, Ryan J, Jacobson NC (2021). Precision assessment of COVID-19 phenotypes using large-scale clinic visit audio recordings: harnessing the power of patient voice. Journal of Medical Internet Research.

[ref-4] Chapman AB, Peterson KS, Turano A, Box TL, Wallace KS, Jones M (2020). A natural language processing system for national COVID-19 surveillance in the US Department of Veterans Affairs. Infectious disease informatics and biosurveillance.

[ref-5] Chatterjee A, Nardi C, Oberije C, Lambin P (2021). Knowledge graphs for COVID-19: an exploratory review of the current landscape. Journal of Personalized Medicine.

[ref-6] Chen S-H, Liao C-C (2011). Are foreign banks more profitable than domestic banks? Home-and host-country effects of banking market structure, governance, and supervision. Journal of Banking & Finance.

[ref-7] Ching T, Himmelstein D, Beaulieu-Jones B, Kalinin A, Do B, Way G, Ferrero E, Agapow P, Zietz M, Hoffman M (2017). Opportunities and obstacles for deep learning in biology and medicine. Journal of the Royal Society, Interface.

[ref-8] Cury RC, Megyeri I, Lindsey T, Macedo R, Batlle J, Kim S, Baker B, Harris R, Clark RH (2022). Natural language processing and machine learning for detection of respiratory illness by chest ct imaging and tracking of COVID-19 pandemic in the us. Radiology: Cardiothoracic Imaging.

[ref-9] De Felice F, Polimeni A (2020). Coronavirus disease (COVID-19): a machine learning bibliometric analysis. In Vivo.

[ref-10] DeSalvo KB, Wang YC, Harris A, Auerbach J, Koo D, OCarroll P (2017). Peer reviewed: public Health 3.0: A call to action for public health to meet the challenges of the 21st century. Preventing Chronic Disease.

[ref-11] Fernandes M, Sun H, Jain A, Alabsi HS, Brenner LN, Ye E, Ge W, Collens SI, Leone MJ, Das S (2021). Classification of the disposition of patients hospitalized with COVID-19: reading discharge summaries using natural language processing. JMIR Medical Informatics.

[ref-12] Guo Y, Zhang Y, Lyu T, Prosperi M, Wang F, Xu H, Bian J (2021). The application of artificial intelligence and data integration in COVID-19 studies: a scoping review. Journal of the American Medical Informatics Association.

[ref-13] Hall K, Chang V, Jayne C (2022). A review on natural language processing models for COVID-19 research. Healthcare Analytics.

[ref-14] Hallak JA, Scanzera A, Azar DT, Chan RV, Paul (2020). Artificial intelligence in ophthalmology during COVID-19 and in the post COVID-19 era. Current Opinion in Ophthalmology.

[ref-15] Inés A, Domínguez C, Heras J, Mata E, Pascual V (2021). Biomedical image classification made easier thanks to transfer and semi-supervised learning. Computer Methods and Programs in Biomedicine.

[ref-16] Islam MM, Karray F, Alhajj R, Zeng J (2021). A review on deep learning techniques for the diagnosis of novel coronavirus (COVID-19). IEEE Access.

[ref-17] John Z (2020). How to fight an infodemic. Lancet.

[ref-18] Khan NA, Albatein J (2021). COVIBOT-an intelligent whatsapp based advising bot for COVID-19.

[ref-19] Lalmuanawma S, Hussain J, Chhakchhuak L (2020). Applications of machine learning and artificial intelligence for COVID-19 (SARS-CoV-2) pandemic: a review. Chaos, Solitons & Fractals.

[ref-20] Legido-Quigley H, Asgari N, Teo YY, Leung GM, Oshitani H, Fukuda K, Cook AR, Hsu LY, Shibuya K, Heymann D (2020). Are high-performing health systems resilient against the COVID-19 epidemic?. The Lancet.

[ref-21] Leswing K (2020). Apple is rejecting coronavirus apps that aren’t from health organizations, app makers say. CNBC. https://www.cnbc.com/2020/03/05/apple-rejects-coronavirus-apps-that-arent-from-health-organizations.html.

[ref-22] Li I, Li Y, Li T, Alvarez-Napagao S, Garcia-Gasulla D, Suzumura T (2020). What are we depressed about when we talk about COVID-19: Mental health analysis on tweets using natural language processing.

[ref-23] Li M, Lang M, Deng F, Chang K, Buch K, Rincon S, Mehan W, Leslie-Mazwi T, Kalpathy-Cramer J (2021). Analysis of stroke detection during the COVID-19 pandemic using natural language processing of radiology reports. American Journal of Neuroradiology.

[ref-24] Locke S, Bashall A, Al-Adely S, Moore J, Wilson A, Kitchen GB (2021). Natural language processing in medicine: a review. Trends in Anaesthesia and Critical Care.

[ref-25] Lybarger K, Ostendorf M, Thompson M, Yetisgen M (2021). Extracting COVID-19 diagnoses and symptoms from clinical text: a new annotated corpus and neural event extraction framework. Journal of Biomedical Informatics.

[ref-26] Neuraz A, Lerner I, Digan W, Paris N, Tsopra R, Rogier A, Baudoin D, Cohen K, Burgun A, Garcelon (2020). AP-HP/Universities/INSERM COVID-19 Research Collaboration; AP-HP COVID CDR Initiative. Natural language processing for rapid response to emergent diseases: case study of calcium channel blockers and hypertension in the COVID-19 pandemic. Journal of Medical Internet Research.

[ref-27] Nham NT, Hong NT (2022). Making the circular economy digital or the digital economy circular? Empirical evidence from the European region. Technology in Society.

[ref-28] Obeid JS, Davis M, Turner M, Meystre SM, Heider PM, O’Bryan EC, Lenert LA (2020). An artificial intelligence approach to COVID-19 infection risk assessment in virtual visits: a case report. Journal of the American Medical Informatics Association.

[ref-29] Ohno-Machado L (2011). Realizing the full potential of electronic health records: the role of natural language processing. Journal of the American Medical Informatics Association.

[ref-30] Pfefferbaum B, North CS (2020). Mental health and the COVID-19 pandemic. New England Journal of Medicine.

[ref-31] Prada Muñoz JL, Sutta Serrano VE (2021). Factores predictores del ingreso a la UCI COVID-19 en dos hospitales públicos del Cusco en junio 2020 a abril 2021. https://hdl.handle.net/20.500.12557/4048.

[ref-32] Schöning V, Liakoni E, Drewe J, Hammann F (2021). Automatic identification of risk factors for SARS-CoV-2 positivity and severe clinical outcomes of COVID-19 using data mining and natural language processing. MedRxiv.

[ref-33] Shorten C, Khoshgoftaar TM, Furht B (2021). Deep learning applications for COVID-19. Journal of Big Data.

[ref-34] Topol EJ (2019). High-performance medicine: the convergence of human and artificial intelligence. Nature Medicine.

[ref-35] Venkatakrishnan A, Pawlowski C, Zemmour D, Hughes T, Anand A, Berner G, Kayal N, Puranik A, Conrad I, Bade S (2021). Mapping each pre-existing conditions association to short-term and long-term COVID-19 complications. NPJ Digital Medicine.

[ref-36] Wang J, Abu-el Rub N, Gray J, Pham HA, Zhou Y, Manion FJ, Liu M, Song X, Xu H, Rouhizadeh M (2021). COVID-19 SignSym: a fast adaptation of a general clinical NLP tool to identify and normalize COVID-19 signs and symptoms to OMOP common data model. Journal of the American Medical Informatics Association.

[ref-37] Yan R, Liao W, Cui J, Zhang H, Hu Y, Zhao D (2021). Multilingual COVID-QA: learning towards global information sharing via web question answering in multiple languages.

